# Simple and sensitive spectrophotometric method for estimating the nitrogen-fixing capacity of bacterial cultures

**DOI:** 10.1016/j.mex.2022.101917

**Published:** 2022-11-07

**Authors:** A. Cordova-Rodriguez, M.E. Rentería-Martínez, C.A. López-Miranda, J.M. Guzmán-Ortíz, S.F. Moreno-Salazar

**Affiliations:** Department of Agriculture and Animal Husbandry, University of Sonora, Road to Kino Bay Km 21, Hermosillo, Sonora, Mexico

**Keywords:** Acetylene reduction assay, Diazotrophic bacteria, ^15^N isotope, NFb, Bromothymol blue

## Abstract

Biological nitrogen fixation (BNF) is a process through which a group of microorganisms called diazotrophs convert unassimilable atmospheric nitrogen into ammonia. In aqueous media, ammonia yields ammonium ions that can be assimilated by microorganisms and plants. To reduce the application of nitrogen fertilizers and their environmental effects, an alternative approach toward sustainable agriculture is the induction of artificial associations between diazotrophs and plants. This has led to increased interest in the search for microorganisms capable of supplying nitrogen to crops. This article presents a simple, economical, and sensitive spectrophotometric method for estimating the BNF capacity of bacteria cultured in a liquid NFb medium, based on the variation of absorbance caused by the change in color of bromothymol blue in the culture medium. The structure and color of this indicator are modified by pH shifts, which depend on the concentration of fixed ammonium ions.

• The nitrogen concentration (estimated from the ammonium in the culture medium) showed a positive correlation (*R*^2^ = 0.984) with the absorbance measured at 610 nm. The regression equation obtained through the origin was *y* = 0.009682140*x*, where *y* is the absorbance and *x* is the nitrogen concentration in the culture medium.
• The methods used at present to measure the efficiency of BNF require expensive equipment, which may not be affordable for many laboratories or companies working in this field.
• This technique can be used for pure bacterial strains and microbial consortia from soil or commercial products.

## Graphical abstract

**Figure:**
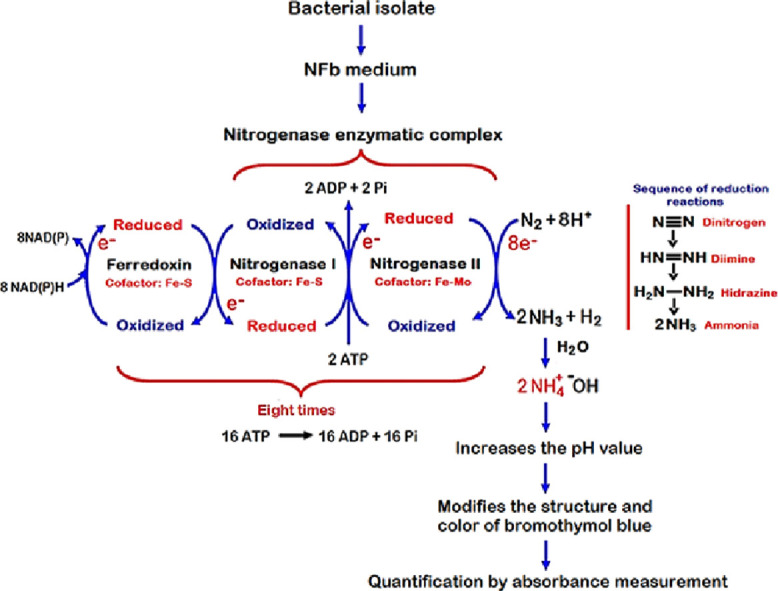

Specifications table

**Table:** 

Subject Area:	Agricultural and Biological Sciences
More specific subject area:	Sustainable agriculture
Method name:	Spectrophotometric method for estimating BNF capacity
Name and reference of original method:	*N.A.*
Resource availability:	*N.A.*

## Introduction

Nitrogen is found in many molecules that are essential for life on Earth. It is a component of amino acids, proteins, and nucleic acids, among other important biomolecules. Molecular nitrogen, dinitrogen (N_2_), which is present in the Earth's atmosphere at levels exceeding 78%, is the primary source for living beings. Despite its abundance, atmospheric nitrogen cannot be assimilated directly by plants and microorganisms; thus, to be used in their metabolic processes, atmospheric nitrogen needs to be converted and fixed in the form of nitrate and/or ammonium ions [1].

Diazotrophs are a group of microorganisms capable of performing biological nitrogen fixation (BNF). An enzyme complex called nitrogenase mediates BNF. Through a series of oxidation–reduction reactions, each nitrogen molecule is converted into two ammonia molecules using eight NAD(P)H molecules, which are previously generated by bacterial catabolism and act as electron and proton carriers. Electrons are first supplied by ferredoxin, after which protons are released. Then, electrons are transferred from the Fe-S centers of nitrogenase I to the Mo-Fe centers of nitrogenase II, activated by ATP. Nitrogenase II reduces the nitrogen in three steps, producing diimine, hydrazine, and ammonia. When solubilized in water, ammonium is converted into ammonium hydroxide, which can be assimilated by plant roots.

Diazotrophs can establish symbiotic or mutualistic associations with various plant species, making nitrogen available to plants. Symbiotic associations involve the formation of root nodules, as observed in legumes and bacteria of the genus *Rhizobium* or in some trees of the *Frankia* sp. Mutualism exists between most plant species and a great diversity of microorganisms belonging to the Archaea and Bacteria domains, which live freely in the plant rhizosphere [2].

**Fig. 1:**
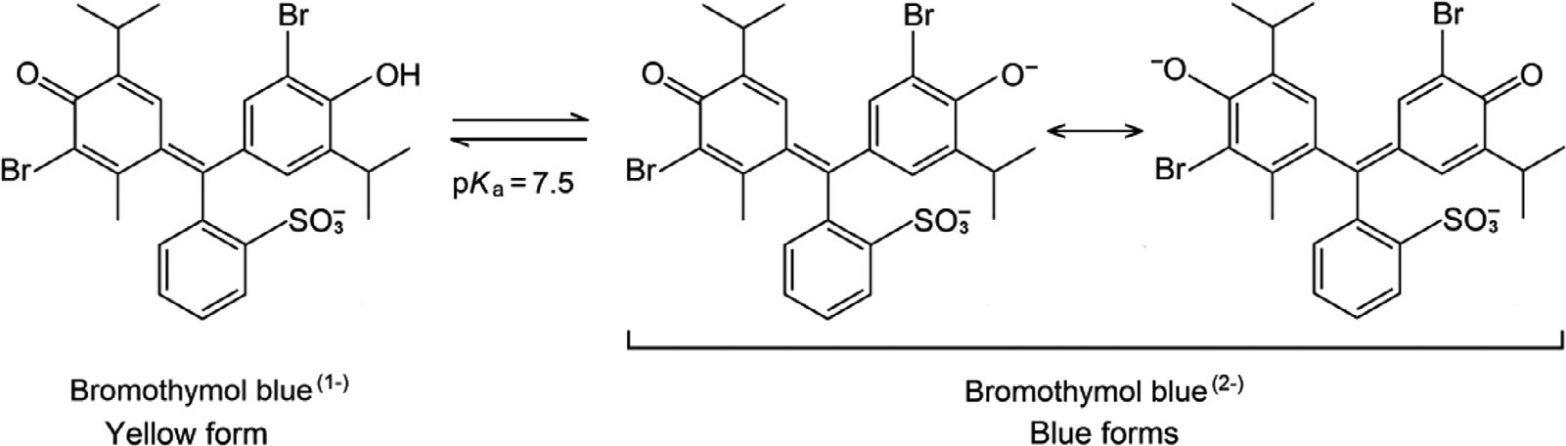
Equilibrium structures of bromothymol blue [4].

A widely used procedure for culturing and isolating diazotrophic bacteria from the rhizosphere, root surface, and/or plant tissues involves the use of the semi-solid nitrogen-free medium, NFb. The color change from green to blue in NFb medium is an indicator of the growth of nitrogen-fixing bacteria [3]. This color change results from the presence of bromothymol blue, whose structure and color depend on the pH. Because of BNF, ammonium ions accumulate in the culture medium and change the pH. Two chemical forms account for the color change in bromothymol blue as a function of pH. In the alkaline medium, the quinoid form, with one negative charge, predominates and shows the yellow color, whereas in the acidic medium, the quinoid-phenolate structure with two negative charges is responsible for the blue color (Fig. 1). The relative concentration of each species determines the different color shades. At pH = 7.5 (p*K*_a_), both reacting species show the same concentration [4].

Since Dilworth (1966) and Schollhorn and Burris (1967) proved that the nitrogenase enzyme complex could reduce acetylene to ethylene with the same demand for NAD(P)H, Mg^++^, and ATP as that required for the reduction of nitrogen to ammonia, the acetylene reduction assay (ARA) has been used to determine the nitrogen-fixing activity of microbial cultures [5]. Other common techniques for the detection of biologically fixed nitrogen consist of the determination of ^15^N concentration after incubation of diazotrophs in an atmosphere containing the said isotope and the digestion and distillation of samples using the Kjeldahl method [6]. However, ARA requires the use of gas chromatography and ^15^N mass spectrometry, which involves the use of equipment that may not be affordable for many researchers in this field. The Kjeldahl method, on the other hand, is laborious and time-consuming, especially when handling large numbers of samples.

Based on the change in color of bromothymol blue due to pH shifts, the use of a spectrophotometric method is proposed to estimate the amount of nitrogen fixed during the growth of diazotrophic bacteria directly in the culture medium, as an economical, simple, and sensitive alternative to the methods currently employed to measure BNF.

## Methodology

### Culture medium composition

Liquid NFb medium (500 mL) composition was as follows: 5 g.L^−1^ malic acid; 0.5 g.L^−1^ K_2_HPO_4_; 0.2 g.L^−1^ MgSO_4_; 0.1 g.L^−1^ NaCl; 0.02 g.L^−1^ CaCl_2_·2H_2_O; 2 mL.L^−1^ micronutrient solution (0.04 g.L^−1^ CuSO_4_·5H_2_O, 0.12 g.L^−1^ ZnSO_4_·7H_2_O, 1.40 g.L^−1^ H_3_BO_3_, 1.0 g.L^−1^ Na_2_MoO_4_·2H_2_O, and 1.175 g.L^−1^ MnSO_4_.H_2_O); 2 mL.L^−1^ bromothymol blue solution (5 g.L^−1^ bromothymol blue in 0.2 N KOH); 4 mL.L^−1^ Fe-ethylenediaminetetraacetic acid (EDTA) solution (16.4 g.L^−1^ Fe-EDTA); 1 mL.*L* ^−^ ^1^ vitamin solution (10 mg biotin and 20 mg pyridoxal-HCl in 100 mL distilled water); and 4.5 g.L^−1^ KOH. Owing to the high pH value, the components were added in the above order to avoid precipitation of Fe and other salts. The pH was adjusted to 6.5 and the medium was sterilized at 121 °C for 15 min [3].

### Standard curve construction

Twenty-three test tubes were prepared, with each containing 2 mL of sterile liquid NFb medium; 0, 0.5, 1, 1.5, 2, 2.5, 3, 3.5, 4, 4.5, 5, 5.5, 6, 6.5, 7, 7.5, 8, 8.5, 9, 9.5, 10, 11, or 12 µL of 0.93 N NH_4_OH solution (standardized using 1.42 N HCl as the secondary standard) were added to each of the test tubes. The total volume of each tube was adjusted to exactly 3 mL using additional culture medium. The concentration of nitrogen in each tube was calculated based on the total volume and the volume of NH_4_OH solution added. Absorbance at 610 nm and pH were measured. This procedure was performed in quadruplicates, and a standard curve of absorbance versus nitrogen concentration was obtained. The experimental correlation of the data was fitted by regression through the origin analysis, and the corresponding equation was calculated using the Excel 2016 statistical analysis tools add-in.

### Use and validation of the standard curve

The amount of nitrogen fixed by *Acinetobacter pitti, A. baumannii, Klebsiella pneumoniae, Enterobacter cloacae*, and *Kosakonia oryzae* was determined using the equation of a fitted line. The previous isolation and identification of these diazotrophic bacteria found in the rhizosphere of *Agave angustifolia* led to the present study being conceived. For each bacterial species, 100 µL of inoculum containing 10^5^ CFU/mL was added to 4.9 mL of sterile liquid NFb medium and incubated at 25 °C for 48 h with constant agitation. Subsequently, the tubes were centrifuged at 5000 rpm and the absorbance of the medium was measured at 610 nm using a Spectronic 20D spectrophotometer. The data were analyzed using one-way ANOVA with Tukey's honestly significant difference (HSD) mean-separation test to interpret significant effects.

## Results

**Fig. 2:**
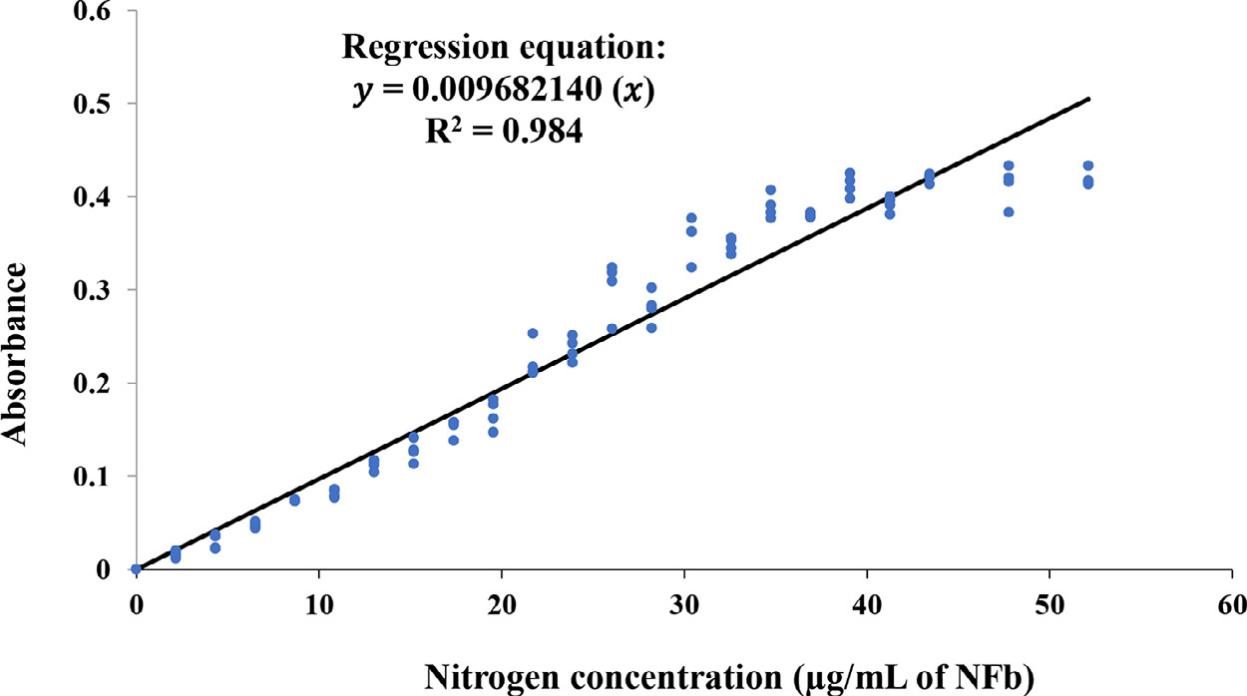
Standard curve of nitrogen concentration in the culture medium versus absorbance.

The nitrogen concentration (estimated from the ammonium ions in the culture medium) showed a positive correlation with the absorbance measured at 610 nm (*R*^2^ = 0.984, *p* < 0.0001, *n* = 89). The fitted regression line through the origin analysis is represented by the equation *y* = 0.009682140x, where *y* is the absorbance and *x* is the nitrogen concentration in the culture medium (Fig. 2).

Table 1 shows the results of the amount of nitrogen fixed by diazotrophic bacteria as determined by the equation of the fitted line of the spectrophotometric method.

**Table 1:** Nitrogen concentration estimated by the spectrophotometric method.

Bacteria	Absorbance	N concentration (µg/mL of NFb)*
*Acinetobacter pitti*	0.178	18.335^d^
*Acinetobacter baumannii*	0.320	33.054^b^
*Enterobacter cloacae*	0.294	30.315^b,c^
*Kosakonia oryzae*	0.344	35.487ª^,b^
*Klebsiella pneumoniae*	0.259	26.797^c^
O*chrobactrum pseudogrignonense*	0.219	22.657^c,d^
*Stenotrophomonas maltophilia*	0.407	42.058^a^

*Values that do not share at least one literal are significantly different (*P* < 0.05).

## Conclusions

• In view of its simplicity, sensitivity, and low cost, the spectrophotometric method developed in this study represents a viable alternative to the traditional methods used to estimate BNF in microbial cultures.
• The spectrophotometric method is capable of detecting significant differences in the nitrogen fixation capacity of different bacterial strains.
• The implementation of this methodology could encourage the search and selection of diazotrophic bacteria (symbiotic, mutualistic, or free-living) in laboratories or companies dedicated to the research and development of environmentally friendly agro-biological products.
• With slight modifications, this technique could also be used to determine the nitrogen-fixing capacity of microbial consortia from soil or commercial samples.

## Data Availability

Data will be made available on request.
